# Supplementary material for: Pyruvate Dehydrogenase Kinase 1 inhibition mediated oxidative phosphorylation enhancement in cartilage promotes osteoarthritis progression
Source: BMC Musculoskelet Disord. 2023 Jul 20;24:597. doi: 10.1186/s12891-023-06585-6 (PMC10357736; doi:10.1186/s12891-023-06585-6)
Supplement: Supplementary file 1 — Additional file 1. [file 12891_2023_6585_MOESM1_ESM.zip › MF.Gsea.1653627316242/GOMF_NOTCH_BINDING.html]

Details for gene set GOMF\_NOTCH\_BINDING[GSEA]

|  || Dataset | 55457\_collapsed\_to\_symbols.cls.cls #OA\_versus\_normal.cls.cls #OA\_versus\_normal\_repos |
| Phenotype | cls.cls#OA\_versus\_normal\_repos |
| Upregulated in class | normal |
| GeneSet | GOMF\_NOTCH\_BINDING |
| Enrichment Score (ES) | -0.5097202 |
| Normalized Enrichment Score (NES) | -1.5021845 |
| Nominal p-value | 0.05631399 |
| FDR q-value | 0.17834449 |
| FWER p-Value | 1.0 |
Table: GSEA Results Summary

  

Fig 1: Enrichment plot: GOMF\_NOTCH\_BINDING      
 Profile of the Running ES Score & Positions of GeneSet Members on the Rank Ordered List

  

| SYMBOL | TITLE | RANK IN GENE LIST | RANK METRIC SCORE | RUNNING ES | CORE ENRICHMENT || 1 | GALNT11 | polypeptide N-acetylgalactosaminyltransferase 11 [Source:HGNC Symbol;Acc:HGNC:19875] | 164 | 0.845 | 0.1202 | No |
| 2 | CUL3 | cullin 3 [Source:HGNC Symbol;Acc:HGNC:2553] | 3480 | 0.204 | -0.1050 | No |
| 3 | CCN3 | cellular communication network factor 3 [Source:HGNC Symbol;Acc:HGNC:7885] | 3924 | 0.162 | -0.1139 | No |
| 4 | SNW1 | SNW domain containing 1 [Source:HGNC Symbol;Acc:HGNC:16696] | 5472 | 0.035 | -0.2285 | No |
| 5 | NCOR2 | nuclear receptor corepressor 2 [Source:HGNC Symbol;Acc:HGNC:7673] | 6497 | -0.042 | -0.3013 | No |
| 6 | SNED1 | "sushi, nidogen and EGF like domains 1 [Source:HGNC Symbol;Acc:HGNC:24696]" | 7029 | -0.082 | -0.3297 | No |
| 7 | DLL3 | delta like canonical Notch ligand 3 [Source:HGNC Symbol;Acc:HGNC:2909] | 7702 | -0.134 | -0.3607 | No |
| 8 | HIF1AN | hypoxia inducible factor 1 subunit alpha inhibitor [Source:HGNC Symbol;Acc:HGNC:17113] | 7815 | -0.142 | -0.3470 | No |
| 9 | CHAC1 | ChaC glutathione specific gamma-glutamylcyclotransferase 1 [Source:HGNC Symbol;Acc:HGNC:28680] | 9853 | -0.307 | -0.4569 | Yes |
| 10 | TCIM | transcriptional and immune response regulator [Source:HGNC Symbol;Acc:HGNC:1357] | 10534 | -0.377 | -0.4505 | Yes |
| 11 | JAG1 | jagged canonical Notch ligand 1 [Source:HGNC Symbol;Acc:HGNC:6188] | 10977 | -0.427 | -0.4176 | Yes |
| 12 | JAG2 | jagged canonical Notch ligand 2 [Source:HGNC Symbol;Acc:HGNC:6189] | 11039 | -0.435 | -0.3539 | Yes |
| 13 | NOTCH4 | notch receptor 4 [Source:HGNC Symbol;Acc:HGNC:7884] | 11441 | -0.495 | -0.3072 | Yes |
| 14 | AAK1 | AP2 associated kinase 1 [Source:HGNC Symbol;Acc:HGNC:19679] | 11842 | -0.558 | -0.2506 | Yes |
| 15 | ADAM17 | ADAM metallopeptidase domain 17 [Source:HGNC Symbol;Acc:HGNC:195] | 11931 | -0.579 | -0.1664 | Yes |
| 16 | DLK2 | delta like non-canonical Notch ligand 2 [Source:HGNC Symbol;Acc:HGNC:21113] | 12524 | -0.760 | -0.0929 | Yes |
| 17 | NOTCH1 | notch receptor 1 [Source:HGNC Symbol;Acc:HGNC:7881] | 12555 | -0.776 | 0.0268 | Yes |
Table: GSEA details [plain text format]

  

Fig 2: GOMF\_NOTCH\_BINDING      
 Blue-Pink O' Gram in the Space of the Analyzed GeneSet

  

Fig 3: GOMF\_NOTCH\_BINDING: Random ES distribution      
 Gene set null distribution of ES for **GOMF\_NOTCH\_BINDING**
